# A Study on the Sub-5 nm Nano-Step Height Reference Materials Fabricated by Atomic Layer Deposition Combined with Wet Etching

**DOI:** 10.3390/mi13091454

**Published:** 2022-09-02

**Authors:** Chenying Wang, Lei Li, Weixuan Jing, Yaxin Zhang, Song Wang, Qijing Lin, Dan Xian, Qi Mao, Yijun Zhang, Duanzhi Duan, Ming Liu, Zhuangde Jiang

**Affiliations:** 1 Collaborative Innovation Center of High-End Manufacturing, Xi’an Jiaotong University, Xi’an 710049, China; 2 International Joint Laboratory for Micro/Nano Manufacturing and Measurement Technology, State Key Laboratory for Manufacturing Systems Engineering, Xi’an Jiaotong University, Xi’an 710049, China; 3 State Key Laboratory of Robotics and Systems, Harbin Institute of Technology, Harbin 150001, China; 4 Electronic Materials Research Laboratory, Key Laboratory of the Ministry of Education, International Center for Dielectric Research, School of Electronic Science and Engineering, Xi’an Jiaotong University, Xi’an 710049, China

**Keywords:** nano-step, atomic layer deposition, step height, roughness

## Abstract

Nano-steps, as classical nano-geometric reference materials, are very important for calibrating measurements in the semiconductor industry; therefore, controlling the height of nano-steps is critical for ensuring accurate measurements. Accordingly, in this study nano-steps with heights of 1, 2, 3 and 4 nm were fabricated with good morphology using atomic layer deposition (ALD) combined with wet etching. The roughness of the fabricated nano-steps was effectively controlled by utilizing the three-dimensional conformal ALD process. Moreover, the relationship between the surface roughness and the height was studied using a simulation-based analysis. Essentially, roughness control is crucial in fabricating nano-steps with a critical dimension of less than 5 nm. In this study, the minimum height of a nano-step that was successfully achieved by combining ALD and wet etching was 1 nm. Furthermore, the preconditions for quality assurance for a reference material and the influencing factors of the fabrication method were analyzed based on the 1 nm nano-step sample. Finally, the fabricated samples were used in time-dependent experiments to verify the optimal stability of the nano-steps as reference materials. This research is instructive to fabricate nano-geometric reference materials to within 5 nm in height, and the proposed method can be easily employed to manufacture wafer-sized step height reference materials, thus enabling its large-scale industrial application for in-line calibration in integrated circuit production lines.

## 1. Introduction

With the development of nanoscale processing technologies, including ultra-precision nanofabrication techniques and nanoelectromechanical systems, the structural dimensions of nanodevices are becoming progressively smaller. The critical dimensions of a geometric structure significantly influence the performance of a nanodevice [1,2,3,4]. Nano-measurement techniques, including the use of measuring devices and methods of measurement, occupy an outstanding position in nanotechnology research. For example, the invention of the scanning tunneling microscope enabled humans to observe, for the first time, the alignment of individual atoms on the surface of matter, and the physical and chemical properties associated with the electronic behavior of a material surface in real time, thus driving a breakthrough in nanotechnology. Therefore, exploration of the measurement accuracy of instruments used for nanometric structures is particularly important, serving as a link between nanodevice performance and nanofabrication processes. Therefore, accuracy needs to be guaranteed when using calibrated nano-reference materials.

Nano-reference materials, as an integral part of the traceability system for nano-measurements, provide a nanoscale standard as a reference for determining the accuracy of other measurements. In general, measured values are compared and traced by nano-reference materials, which play a critical role in quality control during the production process. Accordingly, a reliable reference material is indispensable to guarantee accurate measurements. The nano-step height reference material used for the scale calibration of nano-measurement instruments in the z-direction is a vital component for the length-measure transfer system in nanometrology. 

Currently, the step height reference materials used by most semiconductor companies are manufactured by Very Large Scale Integration Company in the United States, with a step height ranging from 8 nm to 50 μm [5]. Nano-steps with a height of 50 nm and above, used as the national first-class reference material, are fabricated by thermal oxidation in the National Center for Nanoscience and Technology [6]. In general, translating film thickness as a step height is a common fabrication method for nano-step height reference materials. The nano-steps are fabricated using materials such as SiO_2_ or Si_3_N_4_, and these films are deposited on a silicon substrate using a low-pressure chemical vapor deposition technique, a plasma-enhanced chemical vapor deposition technique, and the thermal oxidation process [7,8,9,10,11]. However, the fabrication of sub-5 nm steps using the abovementioned processes has rarely been reported. This indicates that the fabrication of step height reference materials at sub-5 nm level using different techniques needs to be systematically researched.

Focused ion beam technology has been used to fabricate geometric structures by several nanometers in the x-y direction, but with lower accuracy in the z-direction. However, experimental results have proved that the height of nano-steps fabricated using focused ion beam are hard to repeat [12]. Other researchers studied monoatomic steps and found that the height of a monoatomic step was related to the lattice constant of the step material, clearly indicating that the height for this type of reference material is not designable using this method. For example, for SiC and Si monoatomic step reference materials, the single step height values are limited to constant values of 1 and 0.31 nm, respectively [13,14,15]. 

Atomic layer deposition (ALD) is an excellent technique for the growth of ultra-thin film, and it can precisely control the thickness of the deposited film. This ensures a sharp and straight interface between the film and the substrate [16,17,18,19,20,21]. In our previous study, steps with nominal heights of 8, 18, and 44 nm were fabricated by combining ALD with the wet etching process. The step height was calibrated by Physikalisch-Technische Bundesanstaltand. The results were found to be 7.5 ± 1.5, 15.5 ± 2.0, and 41.8 ± 2.1 nm, respectively [22]. Further, the reliability of the fabrication process was also verified. Therefore, this combination is a promising technique to fabricate controllable films with ultra-small surface roughness and a thickness of below 50 nm. Nano-steps of 5, 10, 20 and 40 nm were fabricated using the above methods and then certified as the primary reference materials in China [23]. Nevertheless, due to technical challenges related to the control of surface quality and the ultra-small height of nano-steps, there exist only a few research studies focusing on steps with heights less than 5 nm.

In this study, nano-step height reference materials with heights of 1, 2, 3, and 4 nm were fabricated by growing films with ultra-small surface roughness via ALD and converting the film thickness to step height precisely via wet etching. The minimum height of a nano-step height reference material was then researched. In addition, reasons for the deviations between theoretical and actual minimum height values of the nano-steps were studied. Moreover, the roughness was already unacceptable when the height was at 1 nm. Finally, these fabricated samples were evaluated after one year for height variation to verify the excellent stability of nano-steps as a reference material. Nano-step height reference materials, in particular materials with height of 1 nm, can be used to calibrate step height or film thickness. Future applications are possible in semiconductor processing nodes, transistors, ultra-thin coatings for aerospace aircraft, etc.

## 2. Simulation Analysis

The characteristic values of nano-step height reference materials must satisfy the requirements of accuracy, uniformity and stability, which cannot be achieved without a high-quality step surface. First, the surface quality of Al_2_O_3_ films forming the nano-steps was accurately analyzed via simulation.

Theoretically, the ALD method provides a high-quality film that perfectly duplicates the shape of the underlying substrate, and the surface of the film acquires low roughness because the film is deposited layer by layer via ALD [24,25,26]. This indicates that single-atomic-layer steps can be fabricated using ALD combined with the wet etching process. However, according to another report, the surface roughness of ALD-grown films is larger than that of the substrate [22]. This, in turn, indicates that the surface roughness of the films may not exactly replicate that of the substrate. Therefore, in this study, this phenomenon was analyzed using simulation.

Initially, aluminum oxide was selected as a representative material for nano-steps. Aluminum oxide not only has a high dielectric constant and thermal conductivity but also a strong irradiation damage ability and a resistance to alkali ion penetration. Owing to aluminum oxide having a high stability and an abrasive resistance, it is widely used in manufacturing fields, such as microelectronic devices, electroluminescent devices, optical waveguide devices, and erosion and corrosion resistant coatings [27,28,29]. Furthermore, aluminum oxide films with an excellent blocking performance can be deposited on the surface of monocrystalline silicon as a passivation layer to reduce the defect density. The abovementioned characteristics indicate that aluminum oxide is the best candidate for fabricating nano-steps.

**Figure 1:**
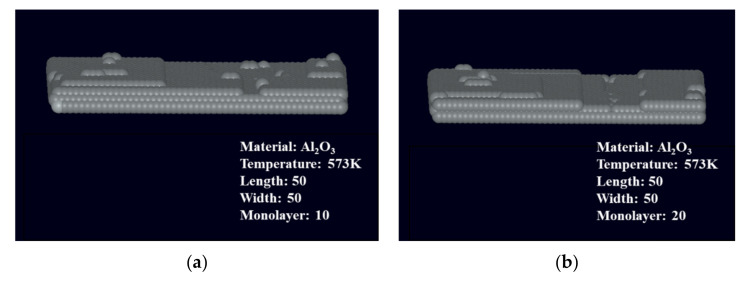

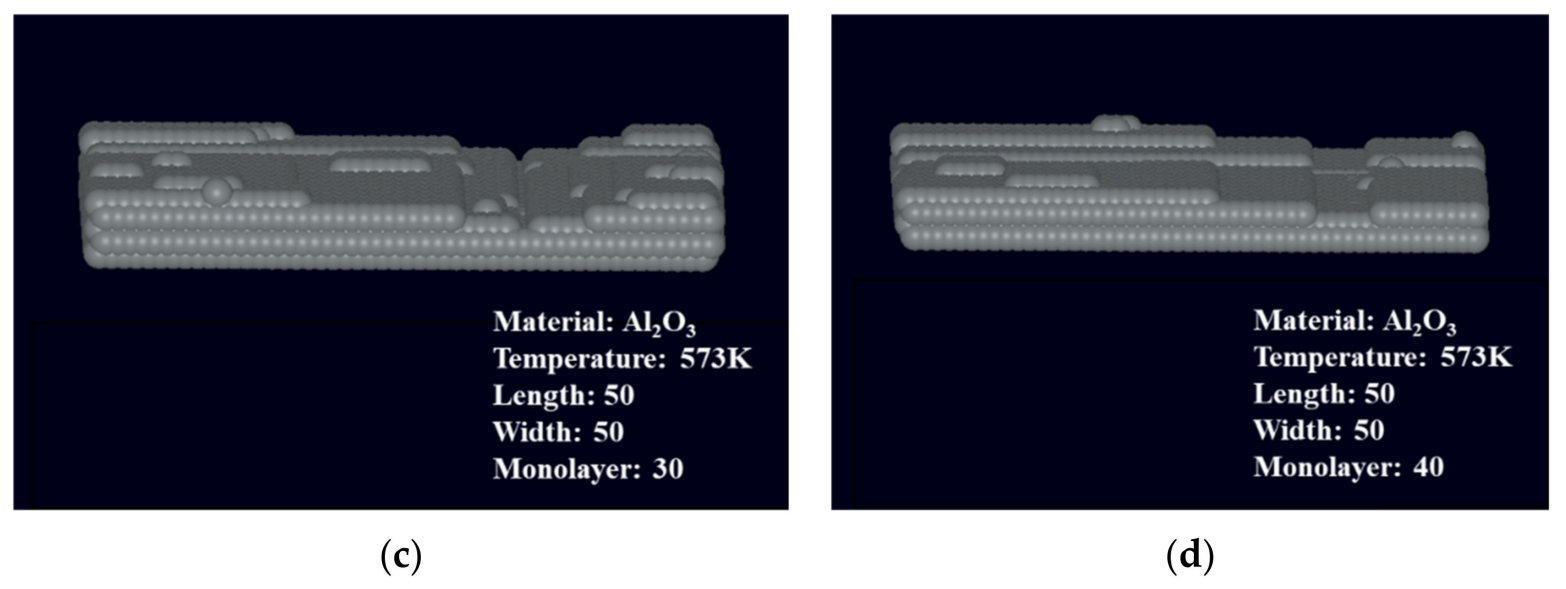
The simulation results for Al_2_O_3_ film growth via ALD based on Monte Carlo simulation. (**a**) 10 cycles. (**b**) 20 cycles. (**c**) 30 cycles. (**d**) 40 cycles.

Subsequently, the simulation of film growth was carried out based on Monte Carlo simulation [30], and the simulated film thicknesses were 1, 2, 3, and 4 nm. Moreover, the film was grown on an exact plane. The typical measured Al_2_O_3_ ALD growth rates are in the range of 1.1-1.2 Å [31,32], and the cycle numbers for generating layers are 10, 20, 30, and 40. After a short nucleation period, the plot of mass change versus the number of cycles was observed to be highly linear. The three-dimensional simulation results are shown in Figure 1. 

The three-dimensional views illustrate that ALD films with thicknesses of around 1–4 nm exhibited particle fluctuations on their surfaces even when the effect of substrate roughness was excluded. These fluctuations consequently increased the surface roughness of the film. The film thickness was converted to step height via wet etching. The surface roughness of the film also affected the uniformity of the step height, thus increasing the measurement uncertainty. When the measurement uncertainty of the step exceeded 50% of the total height, the height value of the step lost its physical significance and could not be used as a standard value for calibration. For this reason, the surface roughness essentially defines the lower limit of nano-step height that can be translated from film thickness. Likewise, a minimum value of height exists for the nano-height reference materials fabricated by integrating ALD and wet etching employed in this study. These needed to be determined by specific experiments.

## 3. Experiment

Based on the simulation results, the reference materials were fabricated and measured by conducting experiments to find the minimum value of step height that could be produced. The steps of Al_2_O_3_ with heights below 10 nm were not visible with an optical microscope due to their poor contrast with the silicon substrate. Therefore, the measurements of step height taken using atomic force microscopy (AFM) required localization. A positioning mark was fabricated on the silicon substrate using the dry etching technique. 

Then, using a remote plasma-enhanced ALD system (R200, Picosun, Masala, Finland), the Al_2_O_3_ film was deposited on the surface of the monocrystalline silicon. The aluminum oxide precursor consisted of trimethyl aluminum (TMA) and water. The formula is:

2Al(CH_3_)_3_ + 3H_2_O = → Al_2_O_3_ + 6CH_4_ (1)

In this study, an experimental plan was designed to achieve the optimal film. Each cycle in ALD included 0.2 s pulses of TMA; 8 s pulses of N_2_; and 0.2 s pulses of H_2_O. Additionally, the carrier gas flow rates for H_2_O and TMA were 200 and 150 sccm, respectively. Fundamentally, the ALD cycle number controls the thickness of the Al_2_O_3_ film, and each cycle generates a monolayer of Al_2_O_3_ film with a thickness of 0.1 nm. The ALD cycle numbers for preparing films of different thicknesses were 10, 20, 30, and 40; accordingly, the thicknesses of the fabricated Al_2_O_3_ films were 1, 2, 3, and 4 nm, respectively.

Next, a layer of photoresist was spin coated onto the Al_2_O_3_ surface. Based on the positioning mark, the morphology of the step structure was duplicated on to the photoresist layer using standard alignment and lithography. 

Then, in a 60 °C water bath environment, the structure was immersed in 60% phosphoric acid to etch the unwanted Al_2_O_3_ film. Following the alumina etch, the photoresist layer covering the structure surface was removed.

**Figure 2:**
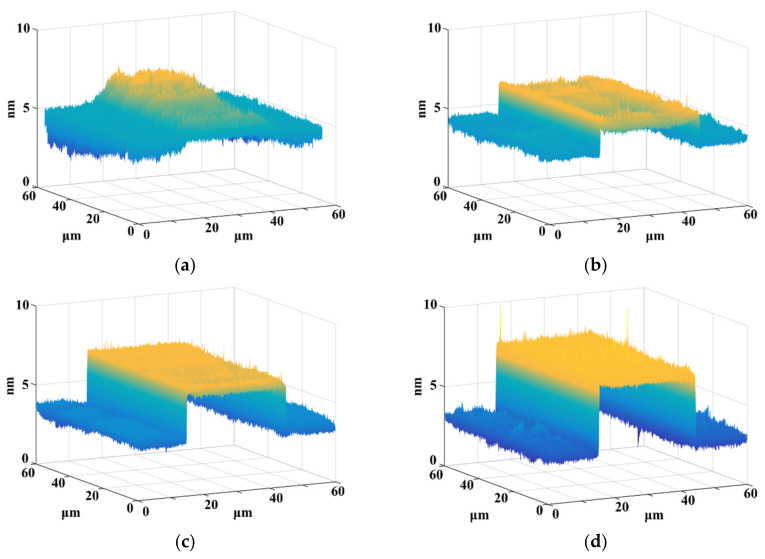
Scanning results of AFM for different heights of nano-steps: (**a**) 1 nm nano-step, (**b**) 2 nm nano-step, (**c**) 3 nm nano-step, (**d**) 4 nm nano-step.

After these processes, Al_2_O_3_ step structures with heights of 1, 2, 3 and 4 nm were obtained and then measured using AFM (INNOVA, Bruker, Karlsruhe, Germany) in tapping mode, as shown in Figure 2. The other specific experimental parameters were discussed in detail in our previous study [22].

## 4. Results and Discussion

**Figure 3:**
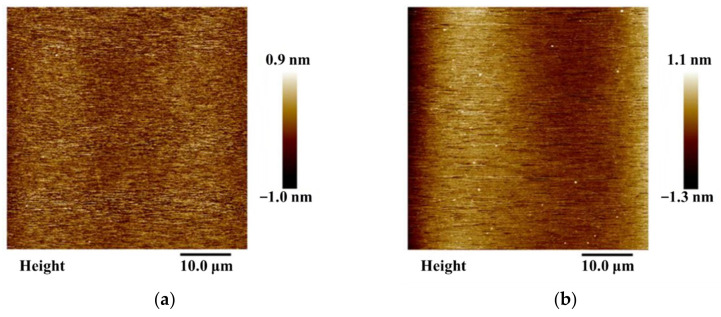

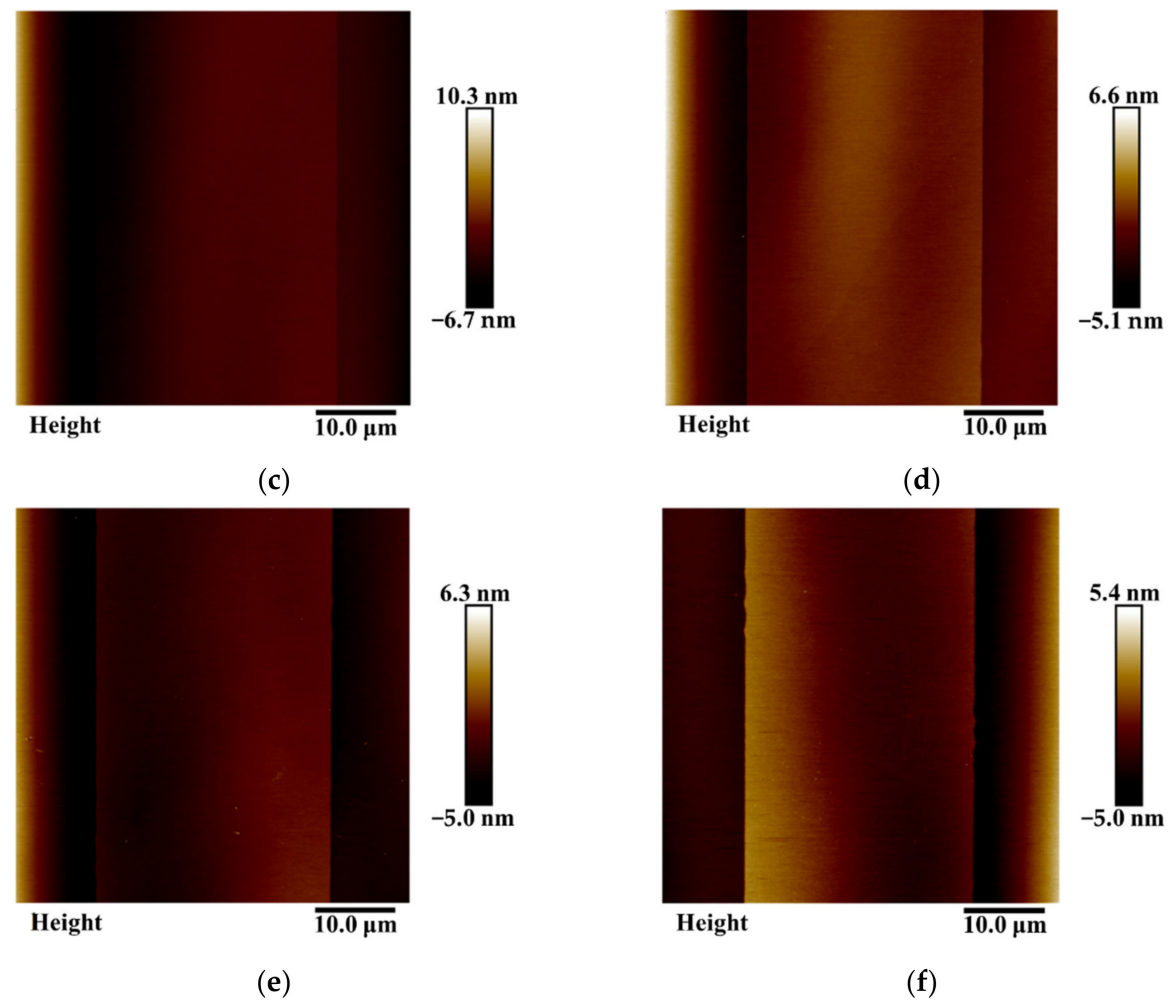
AFM scanning results for surface morphology. (**a**) The surface morphology of monocrystalline silicon measured using AFM. The surface roughness value is R_a_ = 0.161 nm. (**b**) The surface morphology of Al_2_O_3_ film fabricated using ALD. The surface roughness value is R_a_ = 0.165 nm. (**c**) The surface morphology of the nano-step with a height of 1 nm. (**d**) The surface morphology of the nano-step with a height of 2 nm. (**e**) The surface morphology of the nano-step with a height of 3 nm. (**f**) The surface morphology of the nano-step with a height of 4 nm.

To ensure the reliability of the fabrication process and to obtain highly controllable and reproducible nano-step structures, the surface roughness and structural morphology of Al_2_O_3_ films with thicknesses of 1–4 nm grown using ALD and the step surface after wet etching were investigated. The surface morphologies of monocrystalline silicon, Al_2_O_3_ films, and the corresponding nano-steps were characterized using AFM with 512 scanning points, and they are presented in Figure 3.

Figure 3a demonstrates that the surface morphology of monocrystalline silicon is not exactly flat despite the selected substrate material being a hyperplane. The calculated surface roughness of the monocrystalline silicon was R_a_ = 0.161 nm. Furthermore, only slight fluctuations were observed on the surface of the Al_2_O_3_ films, which were much more obvious when compared with the substrate. Figure 3b shows that the roughness of Al_2_O_3_ film was R_a_ = 0.165 nm, which proves that part of the roughness of the Al_2_O_3_ film was caused by ALD.

**Figure 4:**
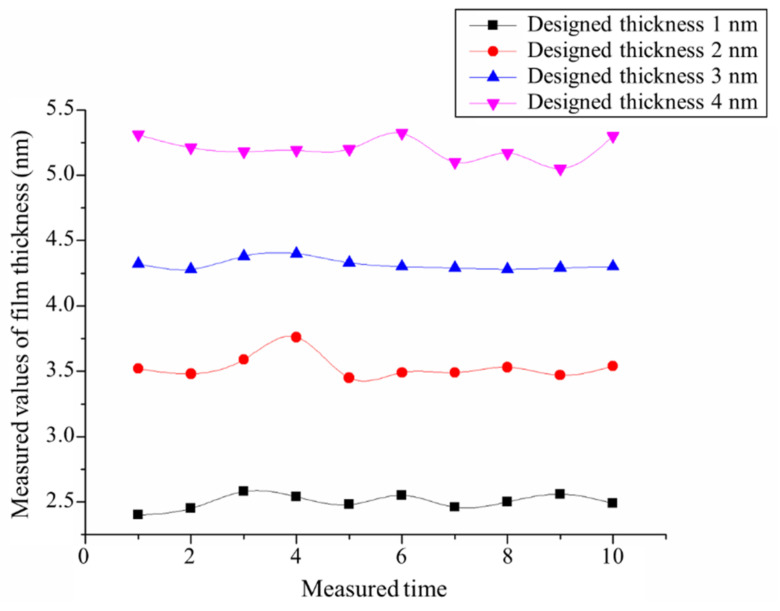
The thickness of Al_2_O_3_ film measured at 10 different points.

The thickness of Al_2_O_3_ film is critical because the final thickness of the film defines the step height. Alpha-se spectral type elliptical polarization apparatus was used to measure the film thickness at 10 different positions. Considering the measurement error of the ellipsometer, Figure 4 illustrates that the data exhibited good consistency; in other words, the thickness of the Al_2_O_3_ film deposited using ALD was uniform and consistent. Thus, ALD provides a guarantee for the fabrication of highly stable and consistent nano-steps. The distance between the adjacent lines was 1 nm, which also indirectly proves the stability and reliability of ALD. The thickness of the film can be controlled precisely by the number of cycles.

The measured thickness values of the Al_2_O_3_ film listed in Table 1 were larger than the designed thickness values. This is attributed to the fact that within the capacity of the ellipsometer there existed a system error, and with an increase in thickness values, the deviation between designed and measured values decreased. Essentially, the film thickness should satisfy the requirement of design after its correction. 

The surface roughness of monocrystalline silicon can be transferred to the Al_2_O_3_ film, owing to the layer-by-layer deposition that can be achieved with ALD. Al_2_O_3_ film acquires a similar morphology to that of the monocrystalline silicon when the Al_2_O_3_ film is assumed to be grown under ideal conditions. However, the simulation results demonstrate the occurrence of fluctuations on the film surface, in addition to the surface roughness being transferred from the monocrystalline silicon substrate. In the experiments, the surface roughness of the Al_2_O_3_ film was dependent on both the substrate morphology and the growth conditions.

When ultra-thin nano-films grow on a silicon substrate, the roughness of the substrate affects the surface roughness of the film. However, tiny surface roughness values of the film itself can be ignored. This indicates that the roughness of Al_2_O_3_ film changes with the film thickness, and the total roughness of the film can be greater than that of its underlying substrate.

**Figure 5:**
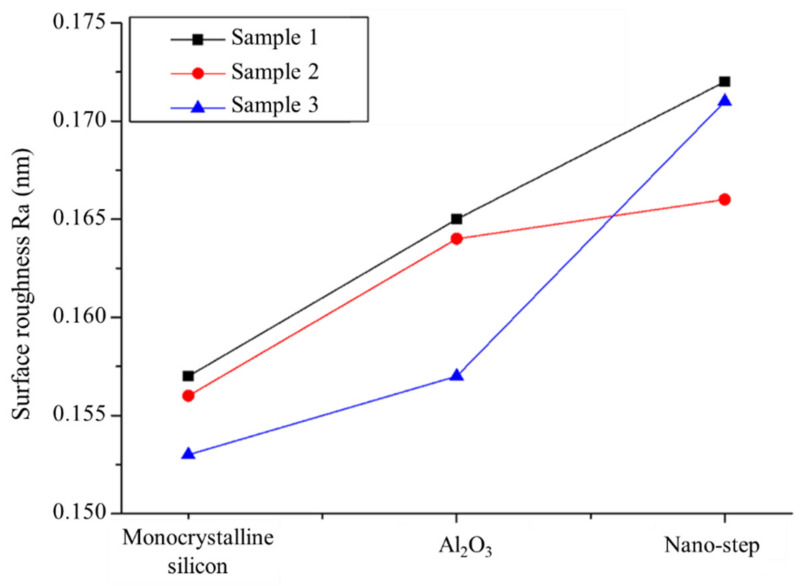
Comparison of surface roughness among monocrystalline silicon, Al_2_O_3_ film, and nano-step.

Figure 5 displays the surface roughness of each monocrystalline silicon, Al_2_O_3_ film, and nano-step for three different samples measured using AFM. The surface roughness of the silicon substrate was below 0.175 nm. This suggests that the experimental substrate was of high quality, which in turn benefited the quality of the sample film surface. After film deposition, the roughness became around 0.155–0.165 nm, which indicates good uniformity of Al_2_O_3_ thickness.

The upper surface roughness of the nano-step increased dramatically following the wet etching process to realize the step structure. The roughness, R_a_, on the surface of the 1 nm step was larger than that of the Al_2_O_3_ film and monocrystalline silicon.

However, R_a_ weakens the local information of surface fluctuation because R_a_ is the arithmetic mean deviation of the measured profile. The height of some particles on the surface of the steps reached 0.7 nm. This indicates that for a step height of 1 nm, the surface quality of the steps was not very good. The uncertainty in the height direction caused by the roughness of the upper and lower surfaces is an important factor that must be considered in the determination of height. This uncertainty was close to half of the height value, reaching the limit of the quality requirement of a reference material.

This was attributed to the introduction of tiny impurities on the nano-step surface during the wet etching process. Moreover, when Al_2_O_3_ film contacted the photoresist and chemical reagent, the surface quality of the film began to deteriorate. These impurities could not be removed by supersonic vibration, and because of these issues, undulation points over 1 nm were counted as gross error and thus excluded when calculating the height of a nano-step.

International algorithms for step height calculation include the histogram method and the least-squares approximation method. The histogram method is a statistical approach that calculates the percentage of height. This method concerns the distance between two points but cannot confirm the condition of the surface, thus requiring a high-quality step surface. Alternatively, the least-squares approximation method fixes two planes to extract the step height. In this study, the latter method was selected to obtain the final height of a step. The method consists of two parallel equations to calculate the altitude difference, and this method avoids calculating the sidewall of the step. The measurement results are presented in Table 2. 

The data presented in the above table indicate that the measurement uncertainty of the step with a designed height of 1.0 nm was already one-half of the measured height, reaching the maximum uncertainty allowed for a reference material. Thus, it was concluded that 1 nm is close to the size limit for the fabrication of step height reference materials with this method. Noteworthy, the measured step height was different from the designed step height because of the deviation between the actual value of film thickness and the designed value of a single cycle. The calculated film thickness for a single cycle was 0.09 nm.

The step reference material needs to be specified with a definitive expiration date; therefore, the stability of the reference material was also tested. The stability of the reference material was defined as the change in the characteristic value of the reference material over time. The longer the stability examination time, the better the stability of the reference material. In this regard, the fabricated Al_2_O_3_ nano-steps were placed in a clean room at room temperature for one year. Then, the nano-steps were measured again using AFM, and the results are presented in Table 2. Clearly, the step height values after one year changed only slightly compared to the values presented in Table 2. Evidently, this indicates that the height of Al_2_O_3_ nano-step fabricated using the proposed method has excellent stability.

## 5. Conclusions

This research proposed a method to fabricate nano-steps with a minimum height of 1 nm by utilizing a combination of the ALD technique and the wet etching process. Compared with typical nano-step materials, such as SiO_2_ or Si_3_N_4_, the materials used for the nano-step fabrication process in this study can be almost any film material that can be deposited by the ALD process. Moreover, the height is not constrained by the step material but instead restricted by the quality of the substrate and film surface. Theoretically, the ALD technique can be understood as growing a monolayer of atoms layer by layer. However, in practice, energy exchanges occur between the absorbed particles and the substrate. These exchanges consume energy that is used to diffuse the particles outward along the vertical direction. Therefore, some particles can diffuse above the surface. The self-diffusion among the particles enables them to move on the surface of the substrate or the film, and the particles continue their motion until they reach a stable position. This movement consequently homogenizes the film growing on top of the substrate. Moreover, with an increase in thickness, an internal stress occurs. The growth mode of particles turns into an island growth mode, and this mode leads to roughness and is the primary reason behind the uncertainty in height. 

Although the surface roughness of the substrate was below 0.2 nm, the minimum roughness of the thin film increased to 0.5 nm after the impact of wet etching. Based on the requirements of reference materials, the uncertainty of measurement should account for less than one-half or even one-third of the step height. However, when the film thickness was less than 1 nm, its surface roughness led to an increase in the measurement uncertainty beyond the requirements. As a result, through the method mentioned in this study, the minimum achievable height of a step was 1 nm, and the height of a single atomic layer film could not be achieved. Moreover, the stability of the nano-step height satisfied the requirement of calibration, which was validated by the one-year-long stability test. In summary, reference materials with a step height of above 1 nm and an extremely small surface roughness can be fabricated through the proposed method for calibrating the measurement error in the z-direction of a measuring instrument. Conversely, this method can be used to fabricate wafer-sized step height reference materials with a step height of above 1 nm for batch production, thus enabling its large-scale industrial application for the in-line calibration in integrated circuit production lines. Undeniably, future systematic explorations are still required to search for new methods for controlling height values of less than 1 nm.

## Figures and Tables

**Table 1:** The thickness of Al_2_O_3_ film measured using the ellipsometer.

Designed Thickness (nm)	1.0	2.0	3.0	4.0
Measured thickness (nm)	2.50 ± 0.05	3.53 ± 0.08	4.32 ± 0.04	5.20 ± 0.08
Difference (nm)	1.50 ± 0.05	1.53 ± 0.08	1.32 ± 0.04	1.20 ± 0.08

**Table 2:** The measured values of nano-step height.

Designed Height (nm)	1.0	2.0	3.0	4.0
Measured height before aging treatment by AFM (nm)	0.8 ± 0.4	1.8 ± 0.5	2.8 ± 0.4	3.7 ± 0.4
Measured height after aging treatment by AFM (nm)	0.9 ± 0.4	1.9 ± 0.5	3.1 ± 0.3	3.8 ± 0.4
